# The impact of energy retrofits on pediatric asthma exacerbation in a Boston multi-family housing complex: a systems science approach

**DOI:** 10.1186/s12940-021-00699-x

**Published:** 2021-02-14

**Authors:** Koen F. Tieskens, Chad W. Milando, Lindsay J. Underhill, Kimberly Vermeer, Jonathan I. Levy, M. Patricia Fabian

**Affiliations:** 1 Department of Environmental Health, Boston University School of Public Health, 715 Albany Street, Boston, MA 02118 USA; 2 Urban Habitat Initiatives Inc, 328A Tremont Street, Boston, MA 02116 USA

**Keywords:** Indoor air quality, Discrete event model, Energy retrofit, Pediatric asthma, Systems science

## Abstract

**Background:**

Pediatric asthma is currently the most prevalent chronic disease in the United States, with children in lower income families disproportionately affected. This increased health burden is partly due to lower-quality and insufficient maintenance of affordable housing. A movement towards ‘green’ retrofits that improve energy efficiency and increase ventilation in existing affordable housing offers an opportunity to provide cost-effective interventions that can address these health disparities.

**Methods:**

We combine indoor air quality modeling with a previously developed discrete event model for pediatric asthma exacerbation to simulate the effects of different types of energy retrofits implemented at an affordable housing site in Boston, MA.

**Results:**

Simulation results show that retrofits lead to overall better health outcomes and healthcare cost savings if reduced air exchange due to energy-saving air tightening is compensated by mechanical ventilation. Especially when exposed to indoor tobacco smoke and intensive gas-stove cooking such retrofit would lead to an average annual cost saving of over USD 200, while without mechanical ventilation the same children would have experienced an increase of almost USD 200/year in health care utilization cost.

**Conclusion:**

The combination of indoor air quality modeling and discrete event modeling applied in this paper can allow for the inclusion of health impacts in cost-benefit analyses of proposed affordable housing energy retrofits.

**Supplementary Information:**

The online version contains supplementary material available at 10.1186/s12940-021-00699-x.

## Introduction

Pediatric asthma is currently the most prevalent chronic disease in the United States. Around 3.2 million children had at least one asthma attack in 2016, resulting in more than 80 thousand hospitalizations [1]. Lanphear et al. demonstrated that 39% of doctor diagnosed pediatric asthma can be attributed to residential risk factors including environmental tobacco smoke (ETS) and indoor allergens [2]. In part due to lower-quality and insufficient maintenance of affordable housing, children and young adults with asthma in low income households suffer from reduced residential indoor air quality (IAQ) [3] and increased exposure to dust allergens, leading to higher counts of serious asthma events and increases in associated healthcare costs [4, 5].

While a push for ‘green’ buildings has initiated retrofits of many existing affordable housing complexes, the explicit inclusion of IAQ, health goals, and subsequent assessment of health outcomes in such retrofits are the exception rather than the rule [6]. In Massachusetts, as in many other states, state law (105 CMR 410.000) requires landlords to provide only the bare minimum of ventilation of a window that can be opened for 4% of its total area. A recent review found that implementing energy saving measures such as air tightening without improving ventilation could result in higher levels of indoor air pollutants and mold growth [7]. Since these indoor air contaminants have both been associated with asthma exacerbations and increasing health costs, any increase in their levels could effectively cancel out energy cost savings [5, 8]. A modeling study in the UK found that energy retrofits when not combined with adequate ventilation could lead to several negative health impacts, including increased asthma exacerbations [9]. Demonstrating the linkages between energy retrofits and IAQ and quantifying the associated health outcomes remains critical to reducing urban health disparities.

Quantifying the link between energy retrofits and health outcomes is not straightforward, as retrofit effects on IAQ and subsequently on residents’ health depend on outdoor levels of pollution and are moderated by specific housing characteristics and a wide range of behavioral patterns (i.e. cooking, smoking, housekeeping) [10, 11]. Additionally, cost-benefit analyses and decision-making related to retrofit investments are complex due to the fact that a large share of the energy savings in subsidized multifamily housing often accrues to the property owner while the health costs are borne by residents. A common approach to study such complex systems is to use systems science techniques to assess how a set of inter-connected sub mechanisms (e.g., residential behavior, airflow, mold growth) together influence specific outcomes (e.g., asthma exacerbation); this is in contrast to traditional techniques that assess the individual impacts of each sub-mechanism [12]. Discrete event model simulations of complex systems, parametrized with epidemiological evidence for individual mechanisms and their interdependencies, provide the opportunity to assess the effects of individual factors within the entire system, and test effects of interventions without the costs normally associated with large-N pre and post intervention assessments [3].

These type of simulation models have been applied to assess the effects of interventions on health outcomes such as malaria [13], diabetes [14], and breast cancer [15]. Their application to studies of environmental exposures and asthma exacerbations has been limited to date. Our research group developed the first systems science model that predicts the daily occurrence of serious asthma events based on exposure to air pollutants from smoking, cooking, and ambient concentrations for individual children while accounting for their personal asthma health, housing, and behavior [10]. This model was used to test the effects of hypothetical retrofits in a low-income housing unit and demonstrated that relatively simple adjustments to standard retrofit practices can reduce the risk of asthma attacks and as a consequence can lead to significant societal cost savings [16].

In this paper, we extend our systems science model to determine the hypothetical pediatric asthma impacts of multiple energy retrofits implemented in 2012 at Castle Square Apartments (CSA), an affordable housing site in Boston, MA. In previous work we modeled how the CSA retrofits differentially impacted IAQ by resident behavior [17]. For example, weatherization alone led to higher indoor concentrations of nitrogen dioxide (NO_2_) and fine particulate matter (PM_2.5_), with greater increases when people smoked inside or cooked more often. In this work, we evaluate how the changes in IAQ can impact asthma outcomes at CSA, identify the main drivers of health outcomes, and show how the impacts would differ for a retrofit lacking ventilation upgrades.

## Methods

### Location

In this study, we utilized a simulation model of the CSA housing complex in the South End neighborhood of Boston, which contains 500 affordable housing units (i.e. units eligible for Housing and Urban Development Section 8 rent subsidies). From 2009 to 2011, energy and green design improvements were implemented as part of a comprehensive Low Income Housing Tax (LIHT) Exempt Bond and 4% LIHT Credit-financed rehabilitation. The property includes four seven-story midrise buildings with 192 units and nineteen four-story low rise buildings with 308 units (stacked townhouses). This study focuses on the low rise buildings, where energy retrofits were expected to reduce energy consumption by 48% and included air sealing, higher efficiency boilers for heat and hot water, HVAC air filtration, kitchen and bathroom exhaust fans, and high efficiency lighting and appliances. In addition, kitchens, bathrooms, and hallways were remodeled using green building materials [17].

### Building characteristics and IAQ

For this study, we modeled units in a typical low-rise building with four stories: stacked townhouses with 8 units per building, four with entries on the ground level and four with entries on the third floor. Each unit has 2-stories, with a living room and kitchen on the lower level and two or three bedrooms and a bathroom on the upper level. Each unit was modeled in a contaminant transport analysis software (CONTAM 3.2.0.3, National Institutes of Standards and Technology, Gaithersburg) as described in detail previously [17, 18]. Briefly, four indoor pollutants were modeled on a daily basis: PM_2.5_ from smoking, cooking, and ambient air; NO_2_ from using the stove for cooking, and infiltration of outside and from neighboring apartments; cockroach allergen (impacted by housekeeping practices and presence of holes in the walls); and mold (predicted based on sustained high relative humidity). Relative humidity was modeled as a contaminant in CONTAM and does not capture potential surface condensation. Modeled resident behavior included cooking frequency and intensity, smoking, window-opening, and housekeeping practices. Hourly outdoor meteorology values (i.e. temperature and relative humidity) were obtained from a typical meteorological year (TMY) dataset (Boston Station 14,739, 1961–1990: TMY2, National Solar Radiation Data Base, National Renewable Energy Laboratory) [19]. Hourly ambient PM_2.5_ and NO_2_ concentrations were obtained from nearby state monitors as described elsewhere [10]. Although CONTAM outputs included separate IAQ outputs for different rooms and zones inside each apartment, we used average daily values for entire apartments for each day.

### Asthma simulation model

We used the core structure of the previously designed discrete event simulation model (DEM) of pediatric asthma [16] to estimate the number of asthma events for children living in CSA, as well as the changes in events due to interventions. Briefly, the model starts with a baseline population of asthmatic children– each living in one of the CSA units and characterized by different housing and behavioral factors such as smoking and cooking, and a randomly assigned baseline lung function expressed as forced expiratory volume percent predicted (FEV1%). FEV1% measures the maximum amount of air a person can forcefully exhale in 1 second, as a percentage of the predicted expected amount based on a person’s characteristics. Daily changes in FEV1% occur in response to exposure to the four pollutants described above (PM_2.5_, NO_2_, cockroach allergen and mold). The changes in FEV1% in turn influence the daily probability of asthma exacerbations and health care utilization (i.e. emergency room visits, hospitalizations, or clinic visits), which are calculated on a daily basis. Asthmatic children are prescribed medication based on their asthma severity category. Severity categories are reassigned annually in the model according to NHLBI guidelines based on each child’s annual average FEV1% (See Fabian et al. [16], for details). Details on model parametrization are provided in Supplementary Information.

The model simulated baseline counts of asthma exacerbations and healthcare utilization outcomes in a cohort of children over a period of 10 years. To ensure statistically robust asthma event prevalence outcomes – particularly for rare health outcomes such as hospitalizations – we simulated a cohort of 10,000 asthmatic children distributed over 1250 blocks of residential units. To capture exposure effects over time all asthmatics were 5 years old at the beginning of the simulation, and aged during a model cycle of 10 consecutive years. The 10,000 asthmatic children of the cohort were assigned a unit in our modeled 8-unit townhouse block (upper or lower level and middle or corner apartment). Infiltration of pollutants between neighboring units in the same block was modeled in CONTAM using parameters typical for the type and age of building. Frequency of cooking using a gas stove (3x per day/ 1x per day) (“Cooking”) and opening windows during summer and fall days (yes/no) (“Windows”) were parameterized using data from a 2013 survey among CSA residents (*n* = 82). The survey included questions on occupant demographics and behavior that was used to parametrize an earlier version of the model and is described in our previous work [17]. Probability of a person in their household smoking indoor (yes or no) (“Smoking”) was based on a survey in public housing in Boston [20]. As this survey was conducted in 2001, before a ban on indoor smoking in Section 8 housing was enacted, this number might be biased. However, the relative values between scenarios as well as the annual healthcare costs are not biased because they are standardized per child per year, and stratified by smoking status. Maintaining an above average or below average level of housekeeping (above/below (“Housekeeping”) was assumed to be split evenly (50/50) (see Table 1).

**Table 1:** Variables used in regression to estimate asthma exacerbation in modeling results

Predictor	Values	Assignment
Base FEV1%	Value from distribution (100, SD = 7) [16]	Random
Retrofit scenarios	Baseline; Retrofit/min; Retrofit/plus	–
Smoking	Yes or No	57% yes
Cooking	Heavy (3x per day) or normal (1x per day)	55% heavy
Windows	Open or closed during summer and fall	75% Open
Housekeeping	Average or above average	50% Average
Apartment type	Corner or middle unit	50% Corner
Apartment Level	Upper or lower level	50% Upper

### Interventions

To assess estimates for direction and strengths of the effects of combinations of different behavior and interventions, we ran a 10 year simulation for the same cohort of 10,000 asthmatic children simulated to live in a CSA apartment for a pre-retrofit baseline scenario and two post-retrofit intervention scenarios, at the end of which asthma outcome prevalence was calculated and compared to the baseline outcomes. The baseline scenario simulated the conditions at CSA prior to the retrofit, while the “retrofit/plus” was created to mimic the energy retrofits that were implemented [17]. The second intervention scenario was added to test the effect of additional ventilation measures. To limit random noise we used a common random sequence to run the simulation model for the same cohort under the same circumstances for baseline and retrofit scenarios [21].

In the baseline scenario 50% of pre-retrofit CSA townhouse units were in good structural condition (i.e. without major holes in the walls or ceilings) and 50% in moderate structural condition (i.e. with major holes in the walls or ceiling which can increase cockroach allergen concentrations as well as air leakage to/from the outdoors). The two retrofit scenarios were: 1) a post-retrofit CSA townhouse in good structural condition (i.e. all holes sealed plus additional air tightening), meeting ventilation standards according to Massachusetts State Law, which included the presence of a bathroom and kitchen window but without kitchen or bathroom exhaust fans (e.g. *Retrofit/min*); 2) a post-retrofit CSA townhouse in good structural condition, meeting ASHRAE 62.2 standards [22], which included installing and operating kitchen and bathroom exhaust fans and operating a mechanical ventilation system to ensure minimum ventilation rates (e.g. *Retrofit/plus*). The two retrofit scenarios were included to assess the health implication of alternative interventions, revealing the difference in effects of recommended ASHRAE ventilation standards (as were implemented in CSA), and mandated minimal ventilation requirements according to Massachusetts State Law. Further discussion on the simulation of these intervention in CONTAM and their effects on indoor air flow and quality can be found in previous work [17].

### Healthcare utilization costs

Total costs include costs of prescribed medication as well as costs for ER visits, hospitalizations, and clinic visits. Treatment costs for clinic visits ($123) were based on the Massachusetts Medical Reimbursement Survey 2015 [23], ER visits ($749) were based on the Medical Expenditure Panel Survey [24], and hospitalizations ($11,932) were based on the Healthcare Cost and Utilization Project based on an average length of 2.4 days [25]. Prescribed medicine costs were based on the cheapest available medicine near CSA on wellrx.com. All costs were adjusted to 2015 dollars based on the Medical Care Consumer Price Index.

### Data output and statistical analyses

Each of the three scenario simulations generated a file with the total number of asthma exacerbations, clinic, emergency room, and hospital visits per child over the 10 year period, and medication prescription and adherence, which were in turn used to calculate the total and average individual health care costs. We compared the difference in average health care costs of the two retrofit scenarios with the baseline scenario.

In order to understand the main housing and tenant characteristic drivers of total asthma outcomes, we built an ordinary least squares linear regression model predicting the annual number of serious asthma events per child. Predictive behaviors included cooking frequency (1x/day or 3x/day), smoking (yes/no), opening windows in the summer and fall (yes/no), and housekeeping (above or below average). The model was controlled for base FEV1%. Additional indicator terms were included for whether the apartment was in the lower or upper level (e.g. stack effect), location in the middle or corner or the building (e.g. increased leakage), and whether the estimate was from a baseline or retrofit scenario. To identify how retrofits could have different effects for different types of behavior, interaction terms were included for retrofit scenarios and smoking, cooking, open windows, and apartment level and location (corner or middle). Base FEV1% was mean centered for easier interpretation of the intercept and regression estimates. All statistical analyses were performed in R version 3.5.3 [26].

## Results

**Fig. 1:**
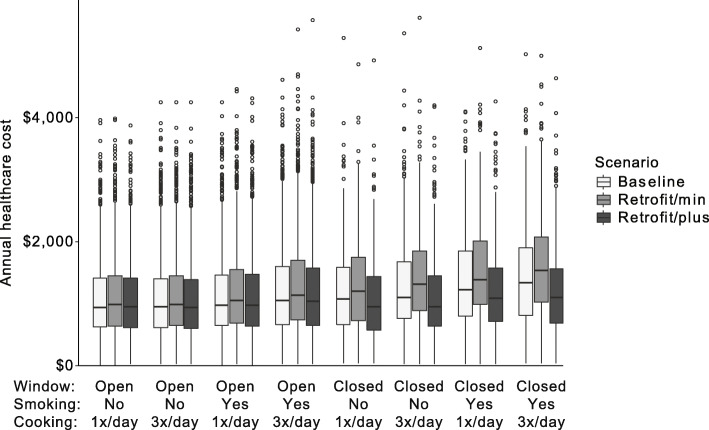
Distribution of average annual healthcare utilization costs per asthmatic per year for different combinations of resident behaviors (cooking frequency (1x/day or 3x/day), smoking (yes/no), opening windows in the summer and fall (yes/no)) for a pre-energy-retrofit Baseline scenario and two post energy-retrofit scenarios (Retrofit/min and Retrofit/plus)

Each of the three model scenarios was simulated for 10,000 children for 10 consecutive years. On average children had 0.86 (±0.34) serious asthma events per year in the Baseline scenario, 0.91 (±0.37) in the Retrofit/min scenario and 0.84 (±0.32) in the Retrofit/plus scenario. The average healthcare cost per asthmatic child was in the Baseline scenario $1164 (±701) per year. Health care costs after retrofit with minimal ventilation (Retrofit/min) was $1253 (±731) per year per child while the Retrofit/plus scenario led to an average cost reduction with $1115 (±675) per year. Health care cost differed substantially for different types of behavior. Figure 1 shows boxplots of the average annual costs per child for different combinations of resident behavior, i.e. cooking frequency (1x/day or 3x/day), smoking (yes/no), and opening windows in the summer and fall (open/closed). For each behavior pattern healthcare costs were the highest in the Retrofit/min scenario, meaning that a retrofit with minimal ventilation efforts led to worse health outcomes on average, regardless of behavior. In both the Retrofit/min and the Baseline scenario, health care costs increased slightly with more intensive cooking and smoking, with greater increases for households that kept windows constantly closed. Annual health care costs for households in the Retrofit/plus scenario were more consistent across different behavior patterns.

**Fig. 2:**
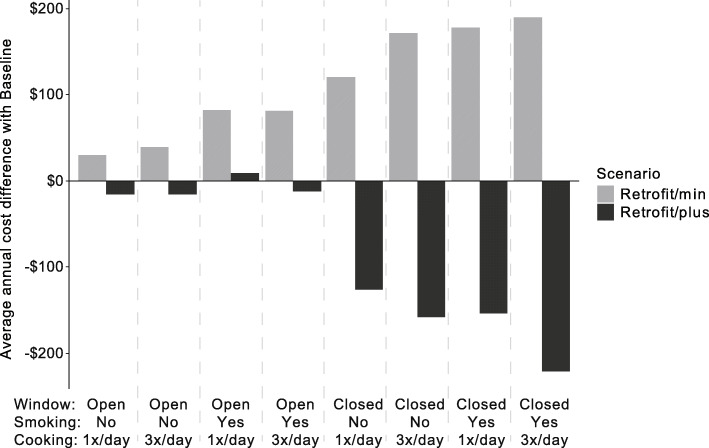
Average annual healthcare utilization cost differences compared with pre-retrofit Baseline scenario for different combinations of resident behaviors. Negative values indicate average savings

The cost differences between the scenarios are highlighted in Fig. 2 below, which shows the average cost difference for all children after the intervention for each behavior pattern. Since the model was run for the exact same cohort with a common random sequence, all modeled cost differences can be attributed to the retrofit intervention. For each type of behavior the Retrofit/min scenario caused an average cost increase as compared to the Baseline scenario, with the difference being larger for closed windows, smoking and 3x/day cooking. In contrast, the Retrofit/plus scenario generally provided cost savings, especially for those who lived in a house with closed windows. Cost savings after retrofits were highest for families smoking inside the house and cooking three times per day with closed windows, with an average saving of over $200 per year. For those with regularly open windows, healthcare cost savings were only marginally different after the Retrofit/plus intervention.

### Serious asthma events predictor models

Results from the OLS regression (Table 2) showed that under the Baseline scenario, a child living in a corner apartment on the ground floor, with normal cooking, no smoking, closed windows and below average housekeeping and a pre-exposure FEV1% of 100 had on average 0.74 serious asthma events per year. The overall fit of the model (R^2^ = 0.19) was relatively low as the simulation model did not include individual characteristics of children or risk factors beyond IAQ exposure.

**Table 2:** Estimates for OLS regression of serious events per asthmatic child per year

*Predictors*	Serious events per asthmatic child per year	
	*Estimates*	*95% CI*
(Intercept)	0.76***	0.74–0.78
Base FEV1%	−0.02***	− 0.02 - -0.02
Windows (open) ^a^	−0.07***	− 0.09 - -0.05
Scenario: Retrofit/min ^a^	0.07***	0.05–0.09
Scenario: Retrofit/plus^a^	−0.08***	− 0.10 - -0.06
Smoking (Yes) ^a^	0.09***	0.08–0.11
Cooking (Heavy) ^a^	0.05***	0.03–0.07
Apartment type (Corner) ^a^	−0.05***	− 0.06 - -0.04
Apartment level (Upper) ^a^	0.02***	0.01–0.03
Housekeeping (Average) ^a^	−0.01	− 0.01 – 0.00
*Interaction terms*		
Windows (open): Retrofit/min	− 0.06***	−0.08 - -0.04
Windows (open): Retrofit/plus	0.09***	0.07–0.11
Smoking (Yes): Retrofit/min	0.03**	0.01–0.05
Smoking (Yes): Retrofit/plus	0.00	−0.02 - 0.02
Cooking (Heavy): Retrofit/min	0.01	− 0.01 - 0.03
Cooking (Heavy): Retrofit/plus	−0.01*	− 0.03 – 0.00
Windows (open): Smoking (Yes)	−0.05***	− 0.06 - -0.03
Windows (open): Cooking (Heavy)	−0.04***	− 0.05 - -0.02
Apartment type (Corner): Apartment level (Upper)	0.05***	0.04–0.07
Observations	30,060	

^a^All predictors were included as binomial factors with Baseline scenario, normal cooking, no smoking, closed windows, and a corner apartment on the ground level as the baseline.

* Significant at *p* < 0.05, ** significant at *p* < 0.01, *** significant at *p* < 0.001

As expected, a higher baseline FEV1% and regularly opening windows predicted a lower number of annual events while smoking and heavy cooking were associated with more annual events per child. The interaction terms of the regression model reveal how different types of behavior result in different health outcomes in the three scenarios. Regularly opening windows decreases the number of annual events per child overall by 0.07. The negative open window - Retrofit/min interaction estimate indicates that regularly opening windows leads to even greater health benefits in the Retrofit/min scenario. In contrast, the installation of ASHRAE compliant ventilation in the Retrofit/plus scenario resulted in opening windows having adverse health effects, while dampening the negative effects of heavy cooking and smoking relative to the Retrofit/min scenario.

## Discussion

By leveraging an existing asthma discrete event simulation tool combined with information from an actual energy retrofit at an affordable housing site in Boston, MA, we estimated the potential impact of two alternative building energy retrofits on asthma outcomes across different resident behavior. The simulation approach was essential to estimate changes in infrequent but costly health outcomes (i.e. asthma hospitalizations) due to building changes, which would not be possible in an intervention field study due to the availability of subjects and/or the prohibitive costs of recruiting a large enough sample size. The results suggest that energy efficiency retrofits that followed ASHRAE 62.2 standards provided a reduction in average serious asthma-related events and associated healthcare costs. However, similar retrofits meeting state mandated minimum ventilation rates lead to an increase of asthma related events and related health care costs. Especially in buildings where occupants smoke tobacco and/or use gas stoves intensively, retrofits in the absence of exhaust fans increased levels of indoor pollutants and thus induced higher number of asthma events. In the extreme (smokers with frequent use of gas stoves who do not open windows), a retrofit with the state mandated minimum ventilation led to asthma-related healthcare costs increasing by an average of almost $200 per year, versus a cost savings of over $200 had the retrofit complied with ASHRAE 62.2 air ventilation standards [22].

Multiple previous studies have shown clear positive effects of green retrofits on health outcomes. Health improvements are attributed to reductions in exposure to allergens, heat, and air pollutants [8, 27–29]. Other studies found less clear positive effects [30, 31] or even observed worse health outcomes for more energy efficient homes [32]. These divergent findings may be related to differential implementation of energy retrofits and the relative balance between air tightening and measures that provide increased ventilation, but the differences among the studies in intervention details, housing types, climates, and other characteristics makes direct comparisons challenging. Our approach, using both IAQ simulation and discrete event modeling, allows for insight on how different combinations of retrofit interventions and behavioral patterns can lead to differential health outcomes. By parameterizing our model using details from an actual energy retrofit and information about resident behavior, we were able to provide interpretable insights.

Our findings reinforced how health effects of interventions to the residential environment are influenced by individual behavior, as well as the complex tradeoffs that occur with multiple sources of asthma triggers. While in the Retrofit/min intervention cockroach allergen was reduced due to building envelope sealing, the air tightening exacerbated the effect of tobacco exposure. This negative effect was essentially eliminated by the increased ventilation in the Retrofit/plus scenario. Additionally, opening windows provides an important mechanism to decrease the effects of smoking and cooking in the Retrofit/min scenario, but this is not an effective measure in the Retrofit/plus scenario, where mechanical ventilation provides improved airflow. Thus, the Retrofit/plus scenario provides overall positive health outcomes with less dependence on individual behavior effects (Fig. 2).

With regards to mechanical ventilation as implemented in Retrofit/plus, our results confirm findings in previous studies that link improvement of ventilation to less asthma related events for children with moderate to severe asthma [31, 33, 34]. Examples from, for instance, California, where building standards require mechanical ventilation in any new home, show significant improvement of residential IAQ and thus occupant health [35].

There are limitations in both the generalizability of our findings and in the insights available from our model platform. Our model only included one building type (four-story multi-family homes) in the urban context of Boston, MA, which may not apply to other settings for multiple reasons. For example, the health implications of window opening clearly depends on ambient air quality relative to the contributions from indoor sources. In our study, we used daily ambient air quality levels measured in Boston with a daily PM_2.5_ average of 12.5 μg/m [3] and daily NO_2_ average of 20.2 ppb. While the model parameters and specific conclusions will differ in settings with higher or lower ambient air pollution, our model can be applied in a variety of settings and with varying ambient concentrations to assess whether opening windows will lead to better IAQ and health outcomes.

In addition, although simulation provided the opportunity to test and compare several different scenarios for the same cohort, both pediatric asthma and retrofits are complex processes that include more aspects than we have included in our simulation model. Apart from hereditary factors, there is ample evidence showing the correlation between asthma exacerbation and stress [36], lifestyle [37], access to health care [38], and neighborhood factors such as crime rate [39, 40], tree pollen [4], and urban greenspace [41]. The rapid growth of environmental data and machine learning techniques can provide ways to include such factors within a systems dynamic modeling framework to assess their respective influence on asthma exacerbation [16]. Such an endeavor could prove especially useful to identify and specifically target social, racial and spatial disparities of pediatric asthma in the US.

Residential behavior in the current model is modeled in a static fashion. Behavior in real life is often more dynamic and complex [37]. We estimated 24-h average concentrations across the entire apartment to approximate exposure. Future work can leverage the multizone modeling capabilities of CONTAM and integrate time activity patterns. For instance exposure to both PM_2.5_ and NO_2_ can be much higher than average during time spent in the kitchen or in the same room as the ETS source, while exposures in the bedroom are lower. Although we modeled the effect of behaviors such as smoking and cooking on the effect of the retrofits on health outcomes, we did not include correlations between occurrences of certain behavior, or the effect the retrofit can have on resident behavior. For instance, a survey that was conducted among CSA residents revealed that one in four residents opened their windows less after retrofits took place than before. In our model, the Retrofit/plus scenario included exhaust fans in kitchens and bathrooms, with a decrease in asthma related events. However, such fans will only provide benefits if they are turned on by residents. In our model, we assumed that these exhaust fans were turned on during showering and cooking.

As is shown in various validation studies, the use of multi-zone airflow models, such as CONTAM, perform well in providing accurate levels of IAQ [42, 43]. In our previous work we evaluated the modeled concentrations of PM2.5, NO2 and cockroach allergen against values published in multiple field studies in Boston multifamily housing and found that there was good concordance [44]. IAQ simulation does still requires a set of assumptions that relate to both physical processes (e.g. infiltration rates, emissions) and human behavior (e.g. frequency of smoking, opening windows). We rely on previously published material for these assumptions, inheriting the uncertainties that come with them.

In spite of these limitations, our model provides insight on the health implications of affordable housing energy retrofits, which often get little attention during large scale renovation of government assisted housing projects. Health disparities related to housing represent an environmental injustice [45, 46], and the elevated risk of health outcomes in affordable housing lead to significant increases in public healthcare spending. The current wave of green energy efficiency retrofits of residential buildings provides an opportunity to reduce both health disparities and public health care spending [47]. Our findings reinforce that the current minimum standards for ventilation in Massachusetts would have been inadequate to protect residents in CSA from exposures that increase pediatric asthma exacerbation, and emphasize the potential benefits of policies such as a ban on indoor smoking in poorly ventilated residential buildings [48].

## Conclusion

With a simulation model that combines detailed IAQ modeling with discrete event simulation of health effects, we showed how the effects of retrofits of affordable housing on pediatric asthma exacerbation vary by attributes of the retrofits and are moderated by behavior of residents. We showed that retrofits could lead to overall better health outcomes and health care cost savings if reduced air exchange due to energy saving air tightening is compensated by mechanical ventilation. IAQ in apartments after retrofits with ventilation meeting ASHRAE standards showed more resilience to individual behavior (i.e. smoking and gas-cooking). However, retrofits that did not meet ASHRAE ventilation standards led to more asthma exacerbation than the pre-retrofit apartments. Our model demonstrates an approach by which health outcomes can be incorporated into cost-benefit analyses of proposed affordable housing retrofits. Future research should focus on expanding the model presented here, by differentiating the type of housing, including neighborhood-specific data for air quality, and introducing other factors related to asthma exacerbation. Validated with real-patient data, such an expanded model would be able to provide estimation of health outcomes and ensure improved health of residents after retrofits.

## Supplementary Information

**Additional file 1.**

## Data Availability

Not applicable.
