# Supplementary material for: The impact of energy retrofits on pediatric asthma exacerbation in a Boston multi-family housing complex: a systems science approach
Source: Environ Health. 2021 Feb 14;20:14. doi: 10.1186/s12940-021-00699-x (PMC7883428; doi:10.1186/s12940-021-00699-x)
Supplement: Supplementary file 1 — Additional file 1. [file 12940_2021_699_MOESM1_ESM.docx]

**The impact of energy retrofits on pediatric asthma exacerbation in a Boston multi-family housing complex: a systems science approach**

**SUPPLEMENTARY MATERIAL**

Koen F. Tieskens PhD
tieskens@bu.edu
Department of Environmental Health, Boston University School of Public Health, 715 Albany Street, Boston, MA, 02118, USA

Chad W. Milando PhD
cmilando@bu.edu
Department of Environmental Health, Boston University School of Public Health, 715 Albany Street, Boston, MA, 02118, USA

Lindsay J. Underhill PhD
lju@bu.edu
Department of Environmental Health, Boston University School of Public Health, 715 Albany Street, Boston, MA, 02118, USA

Kimberly Vermeer MCRP
kim.vermeer@urbanhabitatinitiatives.com
Urban Habitat Initiatives Inc. 328A Tremont Street,
Boston, Massachusetts, 02116, USA

Jonathan I. Levy, ScD,
jonlevy@bu.edu
Department of Environmental Health, Boston University School of Public Health, 715 Albany Street, Boston, MA, 02118, USA

M. Patricia Fabian, ScD
pfabian@bu.edu
Department of Environmental Health, Boston University School of Public Health, 715 Albany Street, Boston, MA, 02118, USA

This supplementary material provides further detail on the parameterization of the CONTAM indoor air modeling and discrete event model simulating the relation between IAQ, FEV1% and serious asthma events. The data tables were adapted from previously published work (1-3).

**Supplement 1. Exposure-related input distributions and equations for discrete event simulation model**

**Table E1. Distributions, values and references for indoor and outdoor pollutant sources used in discrete event simulation model**

| **Indoor pollutant concentrations** | | | | |
| --- | --- | --- | --- | --- |
| PM_2.5_ and NO_2_ | Estimated using regression models, see Supplement 1 | | | |
| Damp house | Dichotomous variable indicating house was “damp”, estimated based on mold index, calculated with differential equations, see section Supplement 1 | | | |
| Cockroach allergen | *Geometric mean* | *Geometric standard deviation* | *Distribution* | *Reference* |
| Bla g 1 in houses… | | | | |
| a) with holes in walls and below average housekeeping | 143.5 U/g | 3.6 | lognormal | raw data from (9) |
| b) with holes in walls and average or >average housekeeping | 42.7 U/g | 6.2 | lognormal | raw data from (9) |
| c) without holes and average or >average housekeeping | 8.2 U/g | 14.6 | lognormal | raw data from (9) |
| Bla g 2 in houses… | | | | |
| a) with holes in walls and below average housekeeping | 691.4 U/g | 8.6 | lognormal | raw data from (9) |
| b) without holes in walls and average or >average housekeeping | 117.3 U/g | 9.0 | lognormal | raw data from (9) |
| c) without holes and average or >average housekeeping | 21.9 U/g | 12.5 | lognormal | raw data from (9) |
| **Other factors** | | | | |
| Indoor multiplication factor (time spent indoors) | | | 0.7 | Table 15-3 of (10) |
| Seasonality factor for “serious” asthma health outcomes | | | (11) | |
| Spring | 1.11 |  | | |
| Summer | 0.60 |  |  |  |
| Fall | 1.23 |  |  |  |
| Winter | 1.05 |  |  |  |
| NO_2_ indoor/outdoor infiltration | 0.58 | Average of infiltration rates reported by (12-15) | | |
| PM_2.5_ indoor/outdoor infiltration | 0.72 | Average of infiltration rates reported by (15-17) | | |

**Supplement 1 equations**

***24-hour indoor NO_2_ and PM_2.5_ concentration equations***

For NO_2_ and PM_2.5_, daily 24-hour average exposures were estimated with regression models developed using the multi-zone simulation software output from CONTAM2.4c (NIST, Gaithersburg, MD, http://www.bfrl.nist.gov/IAQanalysis), an approach described in more detail elsewhere (1). Briefly, within CONTAM, we simulated a low-rise block of eight units of Castle Square apartments using the original building data and drawings (2). A family of 2 adults and 1 asthmatic child was simulated living in each apartment. Sources of NO_2_ included the gas stove used for cooking, the gas oven used for supplemental heat in the winter, and outdoors. Sources of PM_2.5_ included environmental tobacco smoke, cooking, outdoors, and infiltration from neighboring apartments. Detailed schedules of behavior can be found in previously published work (2).

***Mold growth model equations***

Mold growth was calculated using a set of differential equations developed to model mold growth on wooden material (4) which estimate a daily mold index (M) (Table E2).

**Table E2. Description of mold index developed to describe mold growth in wood**


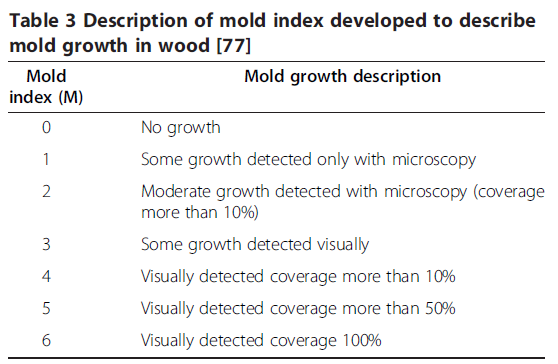


Mold growth was associated with relative humidity with the following equations, taken from (4):

where:

dM/dt = change in mold index (day^-1^)

M = mold index (unitless)

t = time (days)

tm1 = time (weeks) at which mold growth will initiate at constant RH and temp, i.e. M=1

k1,k2 = correction coefficients (unitless), where

if M<1 then

k1 = 1

k2 = 1

if M>=1 then

where

tv = time (weeks) at which there will be visible mold, ie M=3

M_max_ = largest possible value of the mold index at a given

relative humidity and temperature

**Supplement 2. Health outcome-related input distributions and equations for discrete event simulation model**

**Table E3. Distributions, values and references describing lung function parameters, relationships between indoor pollutants and lung function, and lung function and healthcare utilization.**

| **FEV1% distribution & daily variation** | | | | | | |
| --- | --- | --- | --- | --- | --- | --- |
|  | *Mean* | *Standard deviation* | *Distribution* | | *Reference* | |
| Baseline FEV1% | 88.4% | 11.6 | normal | | raw data from HPHI study (n=48) | |
| FEV1% daily difference | 5% | raw data from HPHI study (n = 110 individual week long spirometry sessions, with an average 10 observations/session). Total variability (random + environmental effects) was 10%, we assumed 5% was random variability | | | | |
| **Long term changes in FEV1%** | | | | | | |
| Yearly ΔFEV1% for 5-10 year old asthmatics (18) | | | | | | |
| Without SARE^a^ in last 3 years, compliant and non-compliant | -0.8% |  | | | | |
| With SARE^a^ in last 3 years, compliant | -0.8% |  |  |  |  |  |
| With SARE^a^ in last 3 years, noncompliant | -2.1% |  |  |  |  |  |
| Yearly ΔFEV1% for 11-17 year old asthmatics (18) | | | | | | |
| Without SARE^a^ in last 3 years, compliant and non-compliant | 0% |  | | | | |
| With SARE^a^ in last 3 years, compliant | -0.3% |  |  |  |  |  |
| With SARE^a^ in last 3 years, noncompliant | -1% |  |  |  |  |  |
| **Asthma medication compliance** See Supplement 2 | | | | | | |
| **Coefficients for association between FEV1% and pollutants** | | | | | | |
|  | *Mean* | *Standard error* | *Distribution* | *Reference* | | |
| ΔFEV1% per unit increase in NO_2_ (ppb) | -0.093 % | 0.030 | normal | (19) | | |
| ΔFEV1% per unit increase in PM_2.5_ (ug/m^3^) | -0.077% | 0.032 | normal | (19) | | |
| ΔFEV1% when house classified as “damp” | -10.6% | 4.95 | normal | (20) | | |
| ΔFEV1% per unit increase in log transformed Bla g 1 concentration (U/g) | -0.055% | 0.013 | normal | (5) | | |
| ΔFEV1% per unit increase in log transformed Bla g 2 concentration (U/g) | -0.027% | 0.007 | normal | (5) | | |
|  |  |  |  |  | | |
| **Baseline rates of asthma health outcomes** | | | | | | |
| Serious asthma events | 0.26 events/4 month period | | | | | (7) |
| Hospitalizations | 0.023 per year per asthmatic child | | | | | (21, 22) |
| Emergency room (ER) visits | 0.1 per year per asthmatic child | | | | | (23) |

^a^SARE = severe asthma-related event, defined in our model as a hospitalization or ER visit

**Supplement 2 equations**

***Probability of being prescribed and adhering to taking prescribed asthma medication (i.e. “compliant”)***

We used data from HPHI to estimate the relationship between FEV1% and the probability of being prescribed a controller medication. We used SAS (Proc Logit, version 9.1, SAS Institute Inc., Cary, NC) to calculate the odds of being prescribed asthma medication, and converted the odds ratio to a probability estimate. The resulting probability equation was:

where: P*_med_* is the probability of reporting a controller medication

FEV1% is the baseline lung function value.

The assigned probability was then used in a binomial distribution to transform into a “Yes/No” variable.

***Cockroach allergen equations***

We selected an individual study with all relevant attributes but conducted in adults (asthmatics and non-asthmatics). In this study, Weiss et al. found that log-transformed dust concentrations of Bla g 1 and Bla g 2 were both significantly associated with longitudinal FEV1 decline (ΔFEV1), with multiple linear regression coefficients of -194.14 mL/year and -94.83 mL/year respectively (5). The study did not report functions for asthmatics only, so we used values for the entire population, noting that the relationship between dust concentrations and FEV1 was not appreciably different for the non-asthmatic population than the population as a whole. We converted change in FEV1 (ΔFEV1) to change in FEV1% by dividing ΔFEV1 by FEV1 predicted, where FEV1 predicted was calculated using the NHANES equation below (6), using the average age and height reported in Table 1 of the Weiss study.

where:

age = 57.5 years (5)

height = 174.42 cm (5).

where FEV1predicted = 3.52 L (calculated with previous equation)

ΔFEV1 = -194.14 mL/year for Bla g 1, and -94.83 mL/year for Bla g 2, respectively

***Probability of asthma symptom days***

The frequency of asthma symptoms was characterized in Fuhlbrigge et al (7) (listed in that article’s Figure 1), which shows the number of episode-free days per 4-month period across four categories of FEV1% (<60%, 60-79%, 80-99%, ≥ 100%). An episode-free day was defined as “a day with an asthma diary asthma score of 0, and no report of night awakening, morning and evening peak flow >80% personal best, no albuterol use for symptoms or prednisone use, absence from school as a result of asthma, or physician contact as a result of asthma”. We focused on the number of days with symptoms to be better aligned with our model structure. To convert this into a continuous function of FEV1%, we used the estimated midpoint of each FEV1% (50%, 70%, 90%, and 110%) category and fit the following polynomial expression:

P_symptom_day_ = 2.95 FEV1%^3^ - 6.93FEV1%^2^ + 4.68 FEV1% - 0.27

where

P_symptom_day_ = daily probability of having a day with asthma symptoms as defined above.

FEV1% = forced expiratory volume 1 percent predicted

The equation is valid for values of FEV1% between 0.5 and 1.2.

The assigned probability was then used in a binomial distribution to transform into a “Yes/No” variable.

***Probability of “serious asthma events”***

A similar process was used to fit an equation predicting “serious asthma events”, defined in Fuhlbrigge et al. as oral steroid use, hospitalization, or emergency room visit (7). Table 3 of Fuhlbrigge et al. provides a multivariate regression model including the influence of FEV1% (again in four categories) as well as night awakenings and previous hospitalizations. To convert the reported odds ratios into a probability of a serious asthma event based on a continuous FEV1% scale, we first determined the baseline rate of serious asthma events and converted it to a probability of a serious asthma event. Fuhlbrigge et al reported that their study population had a baseline rate of 0.26 serious asthma events per 4 month period, or approximately 0.0022 events per day (probability of 0.0022). Distributing this rate on a population-weighted basis following odds ratios and population numbers in Table 1 of Fuhlbrigge et al. yields daily event probabilities of 0.0068, 0.0032, 0.0022, and 0.0017 in the four FEV1% categories of decreasing severity. Fitting a polynomial expression to these values leads to a resulting equation of:

P_serious event_ = -0.045FEV1%^3^ + 0.1277FEV1%^2^ - 0.1224FEV1% + 0.0417

where:

P_serious event_ = daily probability of having a serious asthma event

FEV1% = forced expiratory volume 1 percent predicted

The equation is valid for values of FEV1% between 0.5 and 1.2. P_serious event_ was multiplied by a seasonality factor.

The assigned probability was then used in a binomial distribution to transform into a “Yes/No” variable.

***Probability of asthma hospitalization***

We constructed a polynomial equation to predict the daily probability of hospitalization based on FEV1% using the approach described above, with the resulting equation:

P_hosp_ = -0.0013 FEV1%^3^ + 0.0037 FEV1%^2^ - 0.0036 FEV1% + 0.0012

where P_hosp_ includes direct hospitalizations and transfers from the ER to the hospital.

The assigned probability was then used in a binomial distribution to transform into a “Yes/No” variable.

Based on data published in the Fuhlbrigge study, if a child had a hospitalization due to asthma in the previous 12 months , their probability of having a serious asthma event increased (Table 3, (7)). We calculated this multiplicative factor following the same process described above, with the resulting polynomial equation:

O_hospit_ = -45.7FEV1%^3^ + 129.7FEV1%^2^–124.4FEV1% + 42.4

where:

O_hospit_ = increased odds of having a serious asthma event given an asthma hospitalization

in the last 12 months, and was equal to 1 if no hospitalization had occurred.

***Probability of ER visits and oral steroid bursts***

For ER visits, we built a similar equation, where the daily probability of going to the ER is:

P_ER_ = -0.0057 FEV1%^3^ + 0.0162 FEV1%^2^ - 0.0155 FEV1% + 0.0053

Because 8% of ER visits result in hospitalization and are already accounted for in P_hosp_, we multiplied P_ER_ by 0.92 so as not to overestimate ER visits (8).

Oral steroid bursts were estimated by subtracting P_hosp_ and P_ER_ from P_serious event_.

The assigned probability was then used in a binomial distribution to transform into a “Yes/No” variable.

**Supplement 3.**

Table E4 presents the simulated retrofit intervention

| **Retrofit Components** | **Predicted Energy and IAQ impacts** |
| --- | --- |
| Weatherization Measures |  |
| *Air sealing*: Exterior and interior cracks caulked and all doors and windows weather-stripped. *Windows and doors*: Old windows replaced with double-pane glass windows. Sliding glass doors replaced with fiberglass French doors with fixed glass. | Reduction of uncontrolled indoor/outdoor airflows across building envelope to minimize heating and cooling loads and reduce infiltration of outdoor pollutants. |
| Ventilation and Filtration Improvements |  |
| *Ventilation*: New exhaust fans in kitchen and bathroom. Fresh air supply added to HVAC system. | Maintenance of sufficient ventilation, filtration, and fresh air supply to reduce indoor pollutant concentrations. |

**Table E5 Schedules for mechanical and natural ventialtion**

| **Open doors^1^** |  |
| --- | --- |
| Front and back doors | Open for 1 min at 7am, 9am, 1pm, 5pm, 7pm |
| Mechanical door | Door open for 1 min @ 9am |
| **Window-opening^1^** |  |
| Kitchen window | Follows cooking schedule |
| Bathroom window | Follows shower schedule |
| **Exhaust fan operation^2^** |  |
| Kitchen fan   *flow rate=160 cfm* | Follows cooking schedule |
| Bathroom fan  *flow rate=70 cfm* | Follows shower schedule |

### Table E6. Contaminant sources, emission and removal rates, and corresponding schedules.

| **Contaminant and Sources** | **Emission/Removal Rate** | **Reference** | **Schedule** |
| --- | --- | --- | --- |
| **Fine Particulate Matter** |  |  |  |
| Cigarettes (ETS) | 1 mg/min  (10 mg/cig*1 cig/10 min) | Klepeis et al. (2003)(24) | 10 min (1 cigarette) per 1/2 hour from 7:00-20:00 |
| Cooking | 1.56 mg/min | Burk et al. (2001)(25) | Average cooking scenario:  cooking: 8-8:10, 13-13:10, 18:00-18:40; no frying  Heavy cooking scenario:  cooking: 8-8:20 & 13-13:20; frying: 18:00-19:20 |
| Frying | 2.68 mg/min | He (2004)26 |  |
| *ETS PM_2.5_ deposition* | *-0.1/h* | Klepeis & Nazaroff (2006)(27) | All day |
| *Cooking, frying & outdoor PM_2.5_ deposition* | *-0.19/h* | Long et al. (2001)(28) | All day |
| **Nitrogen Dioxide** |  |  |  |
| Cooking (gas stove) | 56 μg/sec | Persily (1998)(29) | Same as PM_2.5_ cooking schedules |
| *Cooking & outdoor NO_2_ decay* | *-0.87/h* | Emmerich & Persily (1996)(30) | All day |
| **Water Vapor** |  |  |  |
| Showering | 670 mg/sec | Persily (1998)(29) | 10-min showers at 6:00, 6:30, & 20:00 (weekdays); 9:00, 9:30, 20:00 (weekends) |
| Cooking | 728.5 mg/sec | Persily (1998)(29) | Same as PM_2.5_ cooking schedule |
| Dishwashing | 83.3 mg/sec | Persily (1998)(29) | 19:00-20:00 |
| Breathing |  |  |  |
| *Children* | Awake: 11.1 mg/sec | Persily (1998)(29) | 6:30-20:00 on weekdays; 8:00-20:00 on weekends |
|  | Sleeping: 6.11 mg/sec |  | 20:00-6:30 on weekdays; 20:00-8:00 on weekends |
| *Adults* | Awake: 15.3 mg/sec | Persily (1998)(29) | 6:00-22:00 |
|  | Sleeping: 8.42 mg/sec |  | 22:00-6:00 |

**Bibliography**

1. Fabian P, Adamkiewicz G, Levy J. Simulating indoor concentrations of NO2 and PM2.5 in multi-family housing for use in health-based intervention modeling. Indoor Air. 2011;2012.
2. Underhill LJ, Fabian MP, Vermeer K, et al. Modeling the resiliency of energy-efficient retrofits in low-income multifamily housing. Indoor Air. 2018;28(3):459-468. doi:10.1111/ina.12446
3. Fabian MP, Adamkiewicz G, Levy JI. Simulating indoor concentrations of NO(2) and PM(2.5) in multi-family housing for use in health-based intervention modeling. Indoor Air. 2011 Sep 13.
4. Hukka A, Viitanen H. A mathematical model of mold growth on wooden material. Wood Science and Technology. 1999;33(6):475-85.
5. Weiss ST, O'Connor GT, DeMolles D, Platts-Mills T, Sparrow D. Indoor allergens and longitudinal FEV1 decline in older adults: the Normative Aging Study. The Journal of allergy and clinical immunology. 1998 Jun;101(6 Pt 1):720-5.
6. Hankinson JL, Odencrantz JR, Fedan KB. Spirometric reference values from a sample of the general U.S. population. Am J Respir Crit Care Med. 1999 Jan;159(1):179-87.
7. Fuhlbrigge AL, Weiss ST, Kuntz KM, Paltiel AD. Forced expiratory volume in 1 second percentage improves the classification of severity among children with asthma. Pediatrics. 2006 Aug;118(2):e347-55.
8. DPHMA. The burden of asthma in Massachusetts. Boston: Department of Public Health2009.
9. Peters JL, Levy JI, Rogers CA, Burge HA, Spengler JD. Determinants of allergen concentrations in apartments of asthmatic children living in public housing. J Urban Health. 2007 Mar;84(2):185-97.
10. EPA. U.S. EPA. Exposure Factors Handbook (External Review Draft) 2009 Update. Washington, DC: U.S. Environmental Protection Agency2009 Contract No.: EPA/600/R-09/052A.
11. Sandel M. 2006-2008 Boston Medical Center Health Net Plan data Quality Improvement study. In: Fabian P, editor. Boston2011.
12. Monn C, Fuchs A, Hogger D, Junker M, Kogelschatz D, Roth N, et al. Particulate matter less than 10 microns (PM10) and fine particles less than 2.5 microns (PM2.5): relationships between indoor, outdoor and personal concentrations. Sci Total Environ. 1997 Dec 3;208(1-2):15-21.
13. Levy JI, Lee K, Spengler JD, Yanagisawa Y. Impact of residential nitrogen dioxide exposure on personal exposure: an international study. J Air Waste Manag Assoc. 1998 Jun;48(6):553-60.
14. Lee K, Levy JI, Yanagisawa Y, Spengler JD, Billick IH. The Boston residential nitrogen dioxide characterization study: classification and prediction of indoor NO2 exposure. J Air Waste Manag Assoc. 1998 Aug;48(8):736-42.
15. Baxter LK, Clougherty JE, Laden F, Levy JI. Predictors of concentrations of nitrogen dioxide, fine particulate matter, and particle constituents inside of lower socioeconomic status urban homes. Journal of exposure science & environmental epidemiology. 2007 Aug;17(5):433-44.
16. Özkaynak H, Xue J, Spengler J, Wallace L, Pellizzari E, Jenkins P. Personal exposure to airborne particles and metals: results from the Particle TEAM study in Riverside, California. J Expo Anal Environ Epidemiol. 1996 Jan-Mar;6(1):57-78.
17. Long CM, Suh HH, Catalano PJ, Koutrakis P. Using time- and size-resolved particulate data to quantify indoor penetration and deposition behavior. Environ Sci Technol. 2001;35(10):2089-99.
18. O'Byrne PM, Pedersen S, Lamm CJ, Tan WC, Busse WW. Severe exacerbations and decline in lung function in asthma. American journal of respiratory and critical care medicine. 2009 Jan 1;179(1):19-24.
19. O'Connor GT, Neas L, Vaughn B, Kattan M, Mitchell H, Crain EF, et al. Acute respiratory health effects of air pollution on children with asthma in US inner cities. The Journal of allergy and clinical immunology. 2008 May;121(5):1133-9 e1.
20. Williamson IJ, Martin CJ, McGill G, Monie RD, Fennerty AG. Damp housing and asthma: a case-control study. Thorax. 1997 March 1, 1997;52(3):229-34.
21. CDC. National Hospital Discharge Survey: 2007 Summary. Atlanta: Centers for Disease Control2010. Report No.: http://www.cdc.gov/nchs/data/nhsr/nhsr029.pdf.
22. CDC. Summary Health Statistics for US Children, National Health Interview Survey 2009. Atlanta: Centers for Disease Control2010. Report No.: http://www.cdc.gov/nchs/data/series/sr_10/sr10_247.pdf.
23. Akinbami LJ. The State of Childhood Asthma, United States, 1980–2005. Atlanta, GA: Centers for Disease Control2006.
24. Klepeis NE, Apte MG, Gundel LA, Sextro RG and Nazaroff WW. Determining size-specific emission factors for environmental tobacco smoke particles. *Aerosol Science & Technology.* 2003;37:780-790.
25. Burke JM, Zufall MJ and Özkaynak H. A population exposure model for particulate matter: Case study results for PM_2.5_ in Philadelphia, PA. *J Expo Sci Environ Epidemiol.* 2001;11:470.
26. He C, Morawska L, Hitchins J and Gilbert D. Contribution from indoor sources to particle number and mass concentrations in residential houses. *Atmos Environ.* 2004;38:3405-3415.
27. Klepeis NE and Nazaroff WW. Modeling residential exposure to secondhand tobacco smoke. *Atmos Environ.* 2006;40:4393-4407.
28. Long CM, Suh HH, Catalano PJ and Koutrakis P. Using time-and size-resolved particulate data to quantify indoor penetration and deposition behavior. *Environ Sci Technol.* 2001;35:2089-2099.
29. Persily AK. A modeling study of ventilation, IAQ, and energy impacts of residential mechanical ventilation. NISTR 6162. National Institute of Standards and Technology. 1998.
30. Emmerich SJ and Persily AK. Multizone modeling of three residential indoor air quality control options. NISTIR 5801. National Institute of Standards and Technology. 1996.
